# Potential Benefits of a Noninvasive Neuromodulation Protocol in Autism Spectrum Disorder with Multiple Comorbidities: A Case Report

**DOI:** 10.3390/pediatric17050092

**Published:** 2025-09-12

**Authors:** Clarissa Aires de Oliveira, Eugenio Luigi Iorio, Foued Salmen Espíndola

**Affiliations:** 1 Biochemistry and Molecular Biology Laboratory, Institute of Biotechnology, Federal University of Uberlandia, Uberlandia 38408-100, Brazil; foued@ufu.br; 2 Program of Post-Graduation in Health Science, Federal University of Uberlandia, Uberlandia 38408-100, Brazil; eugenioluigi.iorio@gmail.com

**Keywords:** Autism Spectrum Disorder, comorbidities, mutation, Radio Electric Asymmetric Conveyer (REAC)

## Abstract

This case report describes a patient (male, 10 years old) with Autism Spectrum Disorder (ASD) and multiple comorbidities, including epilepsy, gastrointestinal and sleep disturbances, and obesity. Whole-exome sequencing (WES) identified two variants of uncertain significance (VUS) in the *GRID2* gene. Mutations in this gene are associated with spinocerebellar ataxia type 18 (SCA18). However, this finding did not correlate with the clinical presentation of the patient. This study evaluates the effects of Radio Electric Asymmetric Conveyer (REAC) stimulation on the cognitive–behavioral dysfunctions of a child with severe ASD and multiple comorbidities. Two stimulation protocols—Neuro Postural Optimization (NPO) and Neuro Psychophysical Optimization (NPPO)—and REAC were performed sequentially. After five weeks of treatment, a 34.9% reduction in total scores on the Autism Treatment Evaluation Checklist (ATEC) and an 8.2% on the Autism Behavior Checklist (ABC) were observed. Assessment of the severity of ASD symptoms using the Childhood Autism Rating Scale (CARS) tool showed less pronounced improvement. The REAC intervention yielded a reduction in Social Relating impairment and an improvement in Sensory/Cognitive Awareness. Further research in this area should employ extended REAC protocols to replicate and amplify clinical responses among individuals with ASD.

## 1. Introduction

Autism spectrum disorder (ASD) is a complex neurodevelopmental condition characterized by altered sensory processing, impairments in social communication, and restricted patterns of behavior [1]. These features vary significantly between individuals and across one’s lifespan. Common ASD-associated comorbidities include intellectual disability [2], gastrointestinal disturbances [3], sleep abnormalities, and epilepsy [4], all of which impact quality of life [5]. The clinical heterogeneity of ASD is associated with multiple genetic mechanisms [6] and different neuroanatomical profiles [7].

The prevalence of gastrointestinal issues such as constipation, diarrhea, and abdominal pain in children with ASD is estimated at 4.2–96.8% [8,9,10]. There are multiple mechanisms for how gut microbes may be involved in the pathophysiology of ASD, including the maternal immune activation model [11,12], the production of neurotransmitters (serotonin and GABA) [13], vagus nerve signaling [11], and the production of short-chain fatty acids [14]. Diet and lifestyle influence the composition and function of the gut microbiota [15]; that is, the community of microorganisms that humans harbor in the gastrointestinal tract [16].

Maternal obesity has been associated with a 1.5-fold increased risk of ASD development in one’s offspring [17,18]. This relationship between maternal obesity and ASD is based on excessive weight gain during gestation [19] and/or maternal metabolic disorders (diabetes) [20]. Mouse pups exposed to a maternal high-fat diet during gestation exhibited ASD-associated phenotypes in cognitive and stereotyped behaviors that were accompanied by central nervous system (CNS) changes [21].

The pathophysiology of ASD remains challenging due to difficulties in consolidating neuroimaging data with molecular and physiological features. In individuals with ASD, an excitation–inhibition (E:I) imbalance may lead to hyperexcitability in cortical circuitry, compromising brain function and social behavior [22,23]. In parallel, histological data have identified altered cortical lamination, along with atypical neuronal migration and changes in the transduction of electrical signaling [24]. These findings demonstrate that the dysfunctions observed in ASD are not limited to a single region of the brain but, rather, involve a disruption in the functioning and integration of multiple neural circuits.

To date, the treatment of ASD typically consists of psycho-educational and behavioral approaches to reduce deficits in social engagement alongside pharmacological interventions for comorbid disorders [25]. However, pharmacotherapy have been associated with long-term side effects, and behavioral intervention may not be feasible [26]. In the absence of causal treatment, it appears crucial to explore the underlying neurobiology and investigate interventions that may have an impact on brain function in ASD [27].

Stimulation with a Radio Electric Asymmetric Conveyer (REAC) has been employed as a non-invasive therapeutic method for a wide range of neurological and psychiatric diseases [28]. Neuroimaging and electrophysiological studies have shown that REAC modulates brain electro-metabolic activity [29] and helps to restore bioelectrical homeostasis [30]. In addition, REAC provides a modulatory effect on cortical function [31] and is easy to apply, as well as being more cost-effective than other non-invasive brain stimulation techniques. In individuals with ASD, REAC has recently been proposed as a promising strategy to reduce symptom severity and improve cognitive functioning [32].

Under this background, we aimed to evaluate the effects of REAC stimulation on cognitive–behavioral dysfunctions in a 10-year-old boy with severe ASD, neurodevelopmental delay, epilepsy, and a compound heterozygous mutation in the *GRID2* gene.

## 2. Case Presentation

I.R.M.S., a 10-year-old male, was diagnosed with ASD at 4 years of age based on a multidisciplinary pediatric neuropsychological evaluation. He was born via cesarean section at 37 weeks’ gestation, following a pregnancy complicated by polyhydramnios. The patient’s Apgar scores were 8 and 9 at 1 and 5 min, respectively. The patient’s birth weight was 2990 g, with a length of 48 cm and an occipital–frontal circumference of 33.5 cm. The patient required oxygen via a nasal cannula for 3 h due to acute respiratory failure, which subsequently resolved. At 2 months of age, he was diagnosed with gastroesophageal reflux disease (confirmed by a swallow study) but maintained growth along the 50th percentile while being treated with ranitidine and omeprazole. Prenatal ultrasound examinations showed no abnormalities. Regarding family history, the patient’s mother was diagnosed with ASD, generalized anxiety disorder, and depression at age 16. His biological father has unspecified cognitive disabilities and epilepsy.

The patient was able to hold up his head at 6 months, sat independently at approximately 8 months, and stood at 13 months. He said his first word at 20 months of age and used sentences at 4 years. He was toilet trained at 6 years of age, but still wets the bed at night. Currently, the patient presents restrictive and repetitive behaviors and is hyper-reactive to sensory stimuli. He also engages in self-injurious behavior, which involves biting and scratching himself when his routine is disrupted. The patient’s gait shows a slight imbalance, as well as a tendency to toe-walk. During the patient’s first 4 years, the mother reported that she did not notice significant difficulties or suspect ASD in her child, attributing his behaviors to his personality traits.

At the age of 3 years, the patient experienced regression in psychomotor development followed by sudden head and eye deviation, as well as tonic–clonic seizures. Several electroencephalogram (EEG) evaluations were conducted, revealing bilateral anterior temporal wave polyspike complexes and epileptic spasms of unknown frequency. The patient required hospitalizations to control the seizures, which were treated with various combinations of valproic acid, topiramate, levetiracetam, and lamotrigine (Table 1).

Brain magnetic resonance imaging (MRI) displayed normal gross anatomy, including the cerebellum. Neurometabolic investigations were unremarkable, with the exception of an intermittently raised serum lactate level (4.2 mmol/L, normal range: 0.70–2.10 mmol/L) early in the course of the disease. The cerebrospinal fluid (CSF) lactate and pyruvate levels were normal. Hemogram, blood sugars levels, serum electrolytes, liver, and renal function were also within normal limits.

At the age of 5 years, whole-exome sequencing (WES) was performed, and two heterozygous variants of uncertain significance (VUS) were identified in the *GRID2* gene. Analysis of the results unveiled no additional mutations associated with ASD and/or epilepsy-related genes. The presence of a heterozygous variant was identified at position c.1472C>T, which has not been previously reported in association with the disease. The second variant, c.2444C>T, causes an amino acid change from Ser to Leu at position 815 (Table 2). Parental screening to determine the carrier status for both mutations could not be performed for financial reasons. Pathogenic variants in the *GRID2* gene are associated with an autosomal recessive form of spinocerebellar ataxia; namely, SCAR18. However, this finding did not correlate with the clinical presentation of the patient.

The patient’s psychiatric history includes anxiety and insomnia managed with sertraline, clonazepam, and melatonin. Ophthalmological examination revealed strabismus and nystagmus. At the gastrointestinal level, the patient suffers from diarrhea and gastroesophageal reflux. Body measurements at 9.5 years were as follows: weight 58 kg, height 145 cm, BMI, 27.6 kg/m^2^ (Z score > 3). His serum 25-hydroxyvitamin D [25(OH)D] level was 17.5 ng/mL.

## 3. Methods

### 3.1. Psychometric Measures

#### 3.1.1. Autism Treatment Evaluation Checklist (ATEC)

The ATEC is a checklist designed to be completed by parents, teachers, and/or primary caretakers of children with ASD. The ATEC includes 4 subscales with 77 items in total: scale I (speech, language, and communication; 14 items, with subscale scores from 0 to 28); scale II (sociability; 20 items, with subscale scores from 0 to 40); scale III (sensory and cognitive awareness; 18 items, with subscale scores from 0 to 36); and scale IV (health, physical condition, and behavior; 25 items, with subscale scores from 0 to 75). Each subcategory of the ATEC scale is scored using a four-point scale as follows: 0 (not a problem), 1 (minor problem), 2 (moderate problem), and 3 (serious problem). The ATEC provides a total score from 0 to 179. For this report, the ATEC was administered twice (pre- and post-REAC).

#### 3.1.2. Childhood Autism Rating Scale (CARS)

Autism severity was assessed using the CARS, 2nd Edition [33]. Total scores can range from a low of 15 to a high of 60; scores below 30 indicate a non-autistic range, scores between 30 and 36.5 indicate moderate autism, and scores from 37 to 60 indicate severe autism [34].

#### 3.1.3. Autism Behavior Checklist (ABC)

The ABC consists of five subscales, including Sensory (9 items, total score of 26), Relating (12 items, total score of 38), Body and object use (12 items, total score of 38), Language (13 items, total score of 31), and Social and Self-help (11 items, total score of 25), comprising a total of 57 items [35].

### 3.2. Genetic Sequencing and Analysis

WES was performed using Twist Bioscience technology (Microgen Genetic Diseases Diagnosis Center). Initially, ~36.5 Mb of consensus coding sequences (targeting >98% of RefSeq and Gencode v. 28 regions obtained from the human genome) were replicated from fragmented genomic DNA using a Twist Human Core Exome Plus kit (Twist Bioscience, South San Francisco, CA, USA). The subsequent library was then sequenced on the Illumina Novaseq NGS (Illumina, San Diego, CA, USA) platform to achieve at least a ×20 reading depth for >98% of the targeted bases [36].

### 3.3. Radio Electric Asymmetric Conveyor—REAC

Neuro Postural Optimization (NPO) and Neuro Psycho Physical Optimization (NPPO) are REAC neuromodulation treatments. The NPO REAC consists of a one-session neurobiological modulation technique that aims to optimize endogenous bioelectrical activity (EBA) and brain electrometabolic activity [29]. The density of the radio-electric current is 7 μA/cm^2^, and the electromagnetic field surrounding the device is approximately 20 μW/m^2^.

For both stimulation protocols, the tip of the metallic REAC asymmetric conveyer probe (ACP) was applied to a specific area of the ear pavilion. After the NPO–REAC treatment, the child underwent a cycle with NPPO, administered in four consecutive sessions, each separated by at least a one-hour interval, for a total of 18 sessions. The REAC NPPO is a neurobiological modulation that improves behavioral functioning and social and environmental interactions [24]. Stimulation was applied once a week for 5 weeks. The REAC treatment parameters were predetermined by the manufacturer and could not be altered by the operator. To administer REAC treatments, we used a BENE mod. 110 (ASMED, Florence, Italy).

### 3.4. Ethics Statement

Written informed consent was obtained from the minor’s legal guardian for the publication of data included in this article. This study adhered to national ethical guidelines and the Declaration of Helsinki.

## 4. Results

### Outcome Measures

**Figure 1:**
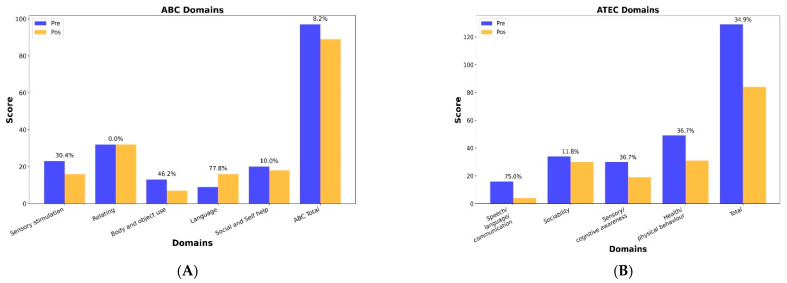
(**A**): Total ABC scores and sub-scores. A comparison between the total ABC scores and sub-scores before and after REAC stimulation; (**B**): Total ATEC scores and sub-scores. A comparison between the total ATEC scores and sub-scores before and after REAC stimulation.

In this report, we compared the changes in ABC and ATEC scale scores before and after intervention with REAC stimulation. The ABC scale results showed that, following REAC intervention, overall scores decreased. Important reductions were observed in four domains: sensory (30.4%), social and self-help (10%), body and object use (46.2%), and language and communication skills (77.8%). The sub-scores in the relating domain showed no changes from before and after REAC stimulation (Figure 1A).

We observed a reduction of 34.9% in the total ATEC score and in the Health and Behavioral Problems (36.7%), Sociability (11.8%), and Sensory/Cognitive Awareness (36.7%) domains. The most pronounced improvement was observed in the Speech/Language/Communication domain, whose score decreased from 16 to 4, corresponding to a 75% reduction (Figure 1B).

No differences were observed in the CARS domains before and after the REAC intervention (Table 3). Adverse effects or sensations related to REAC stimulation (headache, localized pain, tingling, and itching) were assessed through brief questioning. The child did not report any discomfort. Notably, although the patient was not assessed for epilepsy, the parent did report a reduction in epileptic seizures after the REAC treatment.

## 5. Discussion

In this study, we reported the case of a child with ASD carrying a heterozygous mutation in the *GRID2* gene, classified as a variant of uncertain significance (VUS). However, this mutation did not correlate with the clinical presentation, and no brain structural abnormalities were found in the patient. A global impairment of social skills was evidenced by the scores obtained on the ATEC questionnaire, and behavioral problems as reflected in the ABC scale. Moreover, the patient also exhibited multiple comorbidities, including gastrointestinal and sleep disturbances, obesity, and epilepsy. Although the association between epilepsy and ASD is widely reported, little is known about the underlying neurobiological mechanisms. Genes involved in synaptic plasticity [37], structural and functional abnormalities [38] and imbalanced brain excitability [23] appear to be implicated in both conditions.

Genetic and/or environmental risk factors along with immune imbalance have been identified as causative mechanisms underlying the pathogenesis and/or symptomology of co-occurring gastrointestinal problems in ASD [39]. Experimental studies have shown that microbial metabolites can mediate communication between the gut and brain by modulating the excitability of pyramidal neurons [40]. Interventions targeting the microbiota–gut–brain axis, including fecal microbiota transplantation, probiotics, prebiotics, or synbiotics, have shown promising results in improving both the behavioral aspects of ASD as well as comorbid gastrointestinal symptoms [41,42,43].

Previous studies have found an increased risk of overweight or obesity among children and adults with ASD compared to the general population. This association raises important questions about potential contributing factors, such as genetic predisposition, medication effects, dietary patterns, and physical activity levels. Maternal metabolic disorders during pregnancy, such as diabetes, hypertension, and obesity [44], as well as a shorter duration of exclusive breastfeeding, are also associated with a higher risk [45]. Food selectivity in individuals with ASD contributes to a preference for energy-rich foods, sweetened beverages and snacks, and a low intake of vegetables and protein [46]. The use of antipsychotics to reduce disruptive behaviors also contributes to weight gain [47].

Methods for non-invasive brain stimulation (NIBS) have emerged as potentially therapeutic tools for ASD. A previous meta-analysis reported that NIBS improved social-communication symptoms or restricted/repetitive behaviors in individuals with autism [48]. These effects are supported by neuroplasticity and alterations in neuronal excitability [49]. Recent evidence has demonstrated that REAC stimulation can induce modulatory effects on motor coordination and perceptual processing by delivering electromagnetic pulses (2.4, 5.8, or 10.5 GHz). The mechanisms underlying the effects of REAC treatment suggest a preferential modulation of the blood oxygen level-dependent (BOLD) signal in the motor cortex and cerebellum [50].

The REAC intervention significantly improved the symptoms of social-communication and restricted/repetitive behaviors subdomains in our patient. Increased excitability of cells induced by REAC stimulation is thought to enhance blood flow and promote subsequent electrophysiological changes [51]. Moreover, magnetic stimulation increases the cell membrane potential to induce depolarization. Future longitudinal studies are needed to examine how REAC stimulation modulates the topological characteristics of the structural brain network in ASD.

In this study, no adverse events were observed—possibly due to the conservative approach regarding the stimulation intensity used for the pediatric sample. It has been shown that REAC poses minimal risk to children and adolescents with ASD, providing consistent evidence to support the acceptability and tolerability of REAC for other neurodevelopmental disorders [29,30].

There are several limitations to consider in this case study. First, variants in non-coding regions may contribute to the wide range of behaviors observed in ASD. The WES analysis did not reveal any additional mutations directly or potentially associated with epilepsy-related genes. However, we cannot exclude the possibility that other genetic factors not detectable by WES may contribute to the phenotype reported here. Second, the child received antipsychotics, antidepressants, and medications for sleep disorders based on their comorbidities, as well as behavioral therapy and physical exercise intervention programs. These interventions may have influenced the effects of REAC stimulation. A longer follow-up period is needed to determine how long the effects of REAC treatment can be sustained.

## 6. Conclusions

This case report provides evidence for the clinically safe effects of REAC in a patient with severe ASD and multiple comorbidities. The improvement in social and sensory symptoms, as measured by scores on the ABC and ATEC scales, suggests that the combination of REAC protocols may be an important approach to achieve successful outcomes for these individuals. Collaboration between healthcare professionals is essential to develop personalized treatment plans and provide ongoing support.

## Figures and Tables

**Table 1:** Medications administered throughout hospitalization, including class, administration form, dosage, treatment duration (days), and notes on therapeutic purpose or effect.

Class	Medication	Form	Dose	Days	Notes
	Esomeprazole	Oral	Variable	0–end	
Antiemetic	Ondansetron	Oral	Variable	01	
α2A-adrenergic receptor agonist	Clonidine	Oral	Variable	0–12	
Benzodiazepine	Clonazepam	Oral	1–50 mg	0–12	
Antiepileptic	Valproic acid	Oral	8.2 mL	Variable	Substitute for Lamotrigine
Antiepileptic	Lamotrigine	Oral	Variable	0–5	Limited effect
Antiepileptic	Topiramate	Oral	150 mg	0–end	Substitute for Levetiracetam
Antiepileptic	Levetiracetam	Oral	750–2500 mg	0–end	Stopped seizures
Hypnotic	Melatonin	Oral	5 mg	0–end	
Antipsychotic	Aripiprazole	Oral	20 mg	0–end	
Antidepressant	Sertraline	Oral	50 mg	0–end	

**Table 2:** Metrics of GRID2 human variants.

Gene	Transcript	c.DNA	gnomAD Frequency	REVEL	CADD
*GRID2*	NM_001510.4	c.1472C>T	0.00005304	0.19	22
*GRID2*	NM_001510.4	c.2444C>T	0.0024	0.39	29

Abbreviatures: CADD: Combined Annotation Dependent Depletion; REVEL: Rare Exome Variant Ensemble.

**Table 3:** CARS before and after REAC treatment.

	REAC	
CARS Domains	Pre	Post
Personal relationships	3	3
Imitative Behavior	4	4
Emotional Response	4	4
Body Use	3	3
Object Use	3	3
Adaptation to Change	4	3
Visual Response	3	3
Auditory Response	4	4
Taste, Smell, and Touch Response	4	4
Fear or Nervousness	4	4
Verbal Communication	3	3
Nonverbal Communication	4	4
Activity Level	4	3
Level and Consistency of Intellectual Response	3	4
General Impressions	4	4
CARS Total	54	53

Abbreviature: CARS: Childhood autism rating scale.

## Data Availability

The original contributions presented in this study are included in the article. Further inquiries can be directed to the author.

## Abbreviations

The following abbreviations are used in this manuscript:

ABC	Autism Behavior Checklist
ACP	Asymmetric conveyer probe
ASD	Autism Spectrum Disorder
ATEC	Autism Treatment Evaluation Checklist
BOLD	Blood-oxygen-level-dependent
CARS	Childhood Autism Rating Scale
CSF	Cerebrospinal fluid
EBA	Endogenous bioelectric
EEG	Electroencephalography
ID	Intellectual disability
IED	Interictal epileptiform discharges
MRI	Magnetic resonance imaging
NPO	Neuro Postural Optimization
NPPO	Neuro Psycho Physical Optimization
REAC	Radioelectric asymmetric conveyer
VUS	Variants of uncertain significance
WES	Whole exome sequencing
